# Analytical Sensitivity of Eight Different SARS-CoV-2 Antigen-Detecting Rapid Tests for Omicron-BA.1 Variant

**DOI:** 10.1128/spectrum.00853-22

**Published:** 2022-08-08

**Authors:** Meriem Bekliz, Kenneth Adea, Olha Puhach, Francisco Perez-Rodriguez, Stéfane Marques Melancia, Stephanie Baggio, Anna-Rita Corvaglia, Frederique Jacquerioz, Catia Alvarez, Manel Essaidi-Laziosi, Camille Escadafal, Laurent Kaiser, Isabella Eckerle

**Affiliations:** a Department of Microbiology and Molecular Medicine, Faculty of Medicine, University of Geneva, Geneva, Switzerland; b Laboratory of Virology, Division of Infectious Diseases and Division of Laboratory Medicine, Geneva University Hospitals, Geneva, Switzerland; c Division of Prison Health, Geneva University Hospitals & University of Geneva, Geneva, Switzerland; d Geneva Centre for Emerging Viral Diseases, Geneva University Hospitals and University of Geneva, Geneva, Switzerland; e Division of Tropical and Humanitarian Medicine, Geneva University Hospitals, Geneva, Switzerland; f Primary Care Division, Geneva University Hospitals, Geneva, Switzerland; g FIND, Geneva, Switzerland; h Division of Infectious Diseases, Geneva University Hospitals, Geneva, Switzerland; Quest Diagnostics

**Keywords:** Omicron-BA.1 variant, antigen-detecting rapid diagnostic tests, COVID-19, Omicron variant, SARS-CoV-2, variants of concern

## Abstract

The emergence of each novel SARS-CoV-2 variant of concern (VOC) requires investigation of its potential impact on the performance of diagnostic tests in use, including antigen-detecting rapid diagnostic tests (Ag-RDTs). Although anecdotal reports have been circulating that the newly emerged Omicron-BA.1 variant is in principle detectable by Ag-RDTs, few data on sensitivity are available. We have performed (i) analytical sensitivity testing with cultured virus in eight Ag-RDTs and (ii) retrospective testing in duplicates with clinical samples from vaccinated individuals with Omicron-BA.1 (*n* = 59) or Delta (*n* = 54) breakthrough infection on seven Ag-RDTs. Overall, in our analytical study we have found heterogenicity between Ag-RDTs for detecting Omicron-BA.1. When using cultured virus, we observed a trend toward lower endpoint sensitivity for Omicron-BA.1 detection than for earlier circulating SARS-CoV-2 and the other VOCs. In our retrospective study, the detection of Delta and Omicron-BA.1 was assessed in a comparable set of stored clinical samples using seven Ag-RDTs. Four hundred ninety-seven of all 826 tests (60.17%) performed on Omicron-BA.1 samples were positive, compared to 489/756 (64.68%) for Delta samples. In the analytical study, the sensitivity for both Omicron-BA.1 and Delta between the Ag-RDTs was variable. All seven Ag-RDTs showed comparable sensitivities to detect Omicron-BA.1 and Delta in the retrospective study.

**IMPORTANCE** Sensitivity for detecting Omicron-BA.1 shows high heterogenicity between Ag-RDTs, necessitating a careful consideration when using these tests to guide infection prevention measures. Analytical and retrospective testing is a proxy and timely solution to generate rapid performance data, but it is not a replacement for clinical evaluations, which are urgently needed. Biological and technical reasons for detection failure by some Ag-RDTs need to be further investigated.

## INTRODUCTION

The emergence of each novel SARS-CoV-2 variant of concern (VOC) requires investigation of its potential impact on the performance of diagnostic tests in use. SARS-CoV-2 antigen-detecting rapid diagnostic tests (Ag-RDTs) offer quick, inexpensive, and laboratory-independent results at the point of care (1). Although their sensitivity is lower than that of the gold standard method, reverse transcription-PCR (RT-PCR), they enable reliable detection of high-viral-load samples associated with infectious virus presence, making them impactful public health tools (2, 3). However, the majority of Ag-RDT validation studies were performed prior to the emergence of SARS-CoV-2 variants of concern (VOCs) (4).

The VOC Omicron-BA.1 was first reported at the end of November 2021 from South Africa and is characterized by a high number of mutations compared to earlier circulating SARS-CoV-2 (5). The majority of mutations are located in the gene coding for the Spike protein and, according to preliminary data, are associated with considerable escape from neutralization by both disease- and vaccine-derived antibodies and probably also associated with lower vaccine effectiveness (6–10). Current epidemiological data show that Omicron-BA.1 circulation is associated with a steep increase in case numbers as well as an increased risk of reinfection (11).

Beyond the Spike mutations, Omicron-BA.1 also has mutations in the nucleocapsid, which is the target protein of almost all Ag-RDTs. Among the mutations in the nucleocapsid, two (R203K and G204R) have already been described in some SARS-CoV-2 lineages before Omicron-BA.1, in particular Alpha for the mutation R203K and Delta for the mutation G204R. They were linked to increased subgenomic RNA and increased viral loads (12–14). In addition, a deletion (Del31-33) is found in the nucleocapsid of Omicron-BA.1, as well as another mutation, P13L. No information on a potential impact of these mutations on Ag-RDT performance is available so far. Anecdotal reports showed positive detection of Omicron-BA.1-confirmed patient samples by Ag-RDTs, but few experimental data on Ag-RDT sensitivity for Omicron-BA.1 are available.

In this study, we set out to examine 8 of the widely used commercial Ag-RDTs to evaluate their performance for detecting SARS-CoV-2 virus. We sought similar characteristics for the main determinants of rapid test performance, which are viral load, presence of infectious virus, and days post-symptom onset (DPOS).

## RESULTS

### Analytical testing with cultured SARS-CoV-2 isolates.

We have evaluated analytical sensitivity using cultured SARS-CoV-2 Omicron-BA.1 variant, in comparison to previous data obtained on isolates of the other VOCs (Alpha, Beta, Gamma, and Delta) and an early-pandemic (pre-VOC) SARS-CoV-2 isolate (B.1.610) in eight Ag-RDTs. Data on early pandemic SARS-CoV-2, Alpha, Beta, Gamma, and Delta have been published previously but were included here for comparison to Omicron-BA.1 (15, 16). The previously published data referenced in this study were obtained using the same Ag-RDT from the same manufacturer.

Eight Ag-RDTs were used: (i) Panbio COVID-19 Ag Rapid test device (Abbott), (ii) Standard Q COVID-19 Ag (SD Biosensor/Roche), (iii) Sure Status (Premier Medical Corporation), (iv) 2019-nCoV antigen test (Wondfo), (v) Beijng Tigsun Diagnostics Co. Ltd. (Tigsun), (vi) Onsite COVID-19 Ag rapid test (CTK Biotech), (vii) Acon Biotech (Flowflex), and (viii) NowCheck Covid-19 Ag test (Bionote). This list includes all five Ag-RDTs (Panbio, SD Biosensor, Sure Status, Onsite, and Acon) on the WHO Emergency Use Listing (WHO-EUL) and the other tests that are on the waiting list for WHO-EUL approval (17).

**FIG 1:**
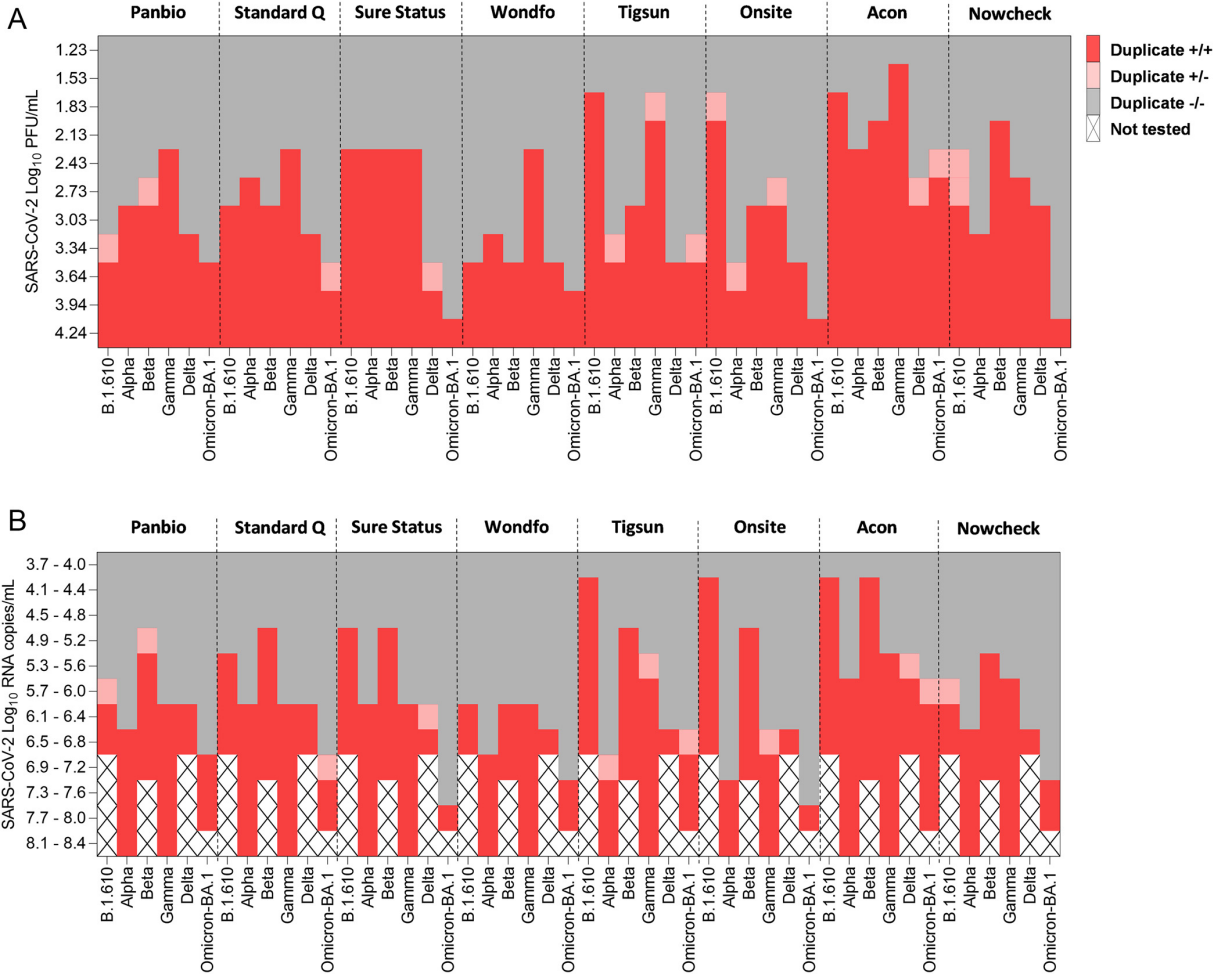
Heatmap based on log_10_ PFU per milliliter (A) and on RNA viral load ranges (B) for analytical sensitivity of eight Ag-RDTs with an early-pandemic SARS-CoV-2 isolate (B.1.610) and the VOCs Alpha, Beta, Gamma, and Delta in comparison to Omicron-BA.1. Note that analytical sensitivities for early-pandemic SARS-CoV-2 B.1.610, Alpha, Beta, Gamma, and Delta have already been published before but were added here for consistency reasons and better interpretability of the data on Omicron-BA.1 (15, 30).

When assessed by infectious virus titer (PFU per milliliter) (Fig. 1A), analytical sensitivity to detect Omicron-BA.1 was lower than that for the other VOCs in most of the tests evaluated. Two tests, Acon and Tigsun, showed a slightly higher sensitivity for Omicron-BA.1 than for Delta, but for these tests, sensitivity for both Delta and Omicron-BA.1 was lower than that for the other VOCs and pre-VOC SARS-CoV-2. The same pattern of the lowest sensitivity for Omicron-BA.1 compared with the other VOCS was confirmed when assessing RNA copy numbers (Fig. 1B). Overall sensitivity and specificity for the Omicron-BA.1 isolate varied between Ag-RDTs, with the highest sensitivity of detection observed with the Acon Ag-RDT and lowest sensitivity of detection observed with Sure Status, Onsite, and NowCheck. In our analytical study, considerable heterogenicity between Ag-RDTs has been found for detecting Omicron-BA.1.

### Sensitivity testing in patient specimens.

In addition to this analytical work, we have tested seven of the eight Ag-RDTs with original patient specimens as a retrospective sensitivity study with 113 nasopharyngeal specimens of confirmed Omicron-BA.1 (*n* = 59) or Delta (*n* = 54) breakthrough infections in vaccinated individuals during the first 5 DPOS. Overall, the Omicron-BA.1 and Delta patients’ specimen collections did not significantly differ in RNA viral load, DPOS, or number of specimens with infectious virus (Table 1).

**TABLE 1:** Characteristics of clinical specimens

Characteristic	Omicron-BA.1 (*n* = 59)	Delta (*n* = 54)	*P* ^*a*^
Log_10_ SARS-CoV-2 copies, mean (SD)	7.8 (0.7)	8.0 (0.8)	0.113
DPOS, mean (SD)	2.6 (1.4)	2.4 (1.6)	0.598
Presence of infectious virus, *n* (%)	46 (78.0%)	39 (72.2%)	0.481

a *P* values for simple linear regressions (log_10_ SARS-CoV-2 copies, DPOS) and simple logistic regressions (presence of infectious virus) are reported.

**FIG 2:**
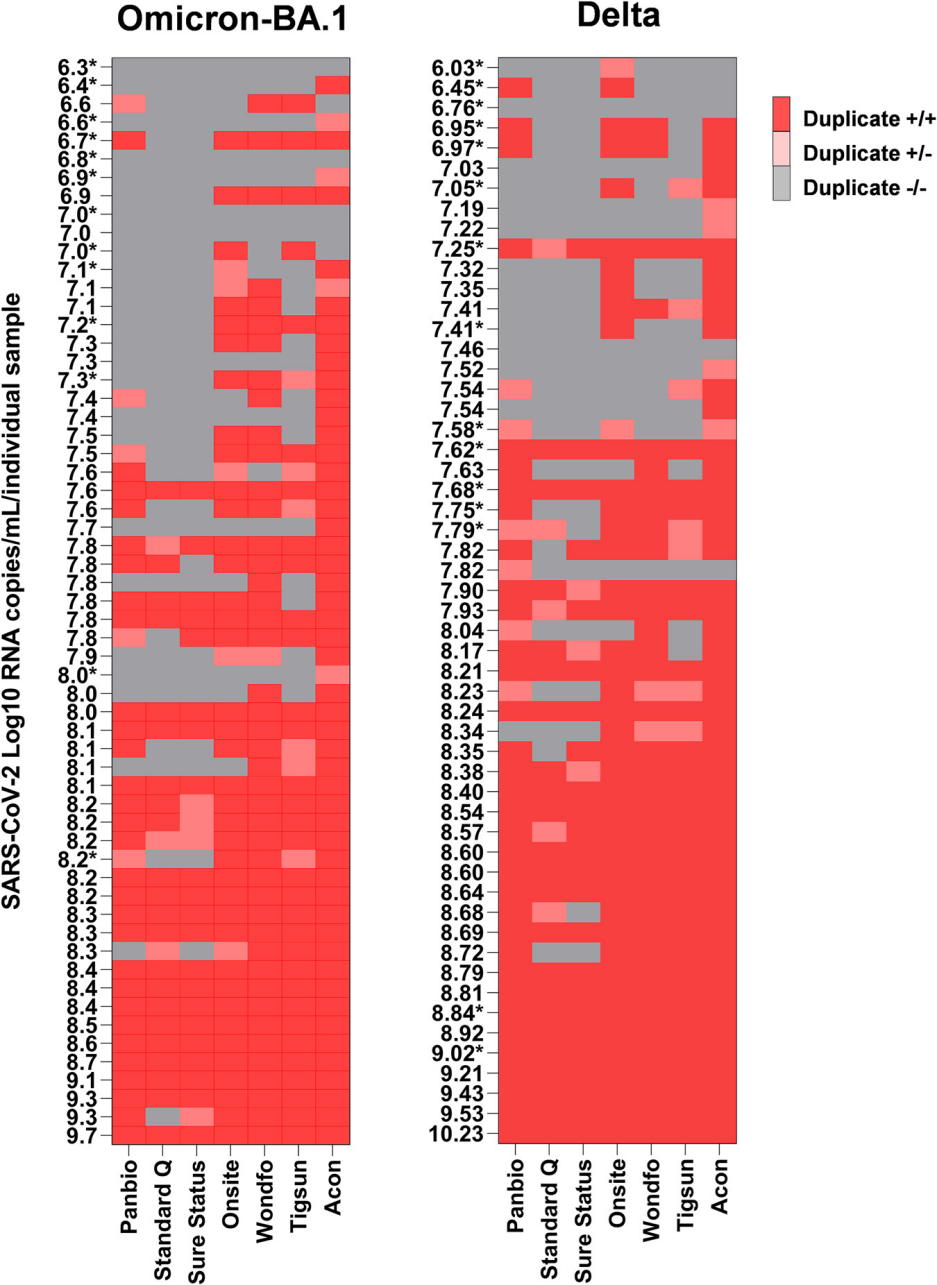
Heatmap of retrospective testing of original nasopharyngeal patient swab specimens from Omicron-BA.1 (*n* = 59) and Delta (*n* = 54) breakthrough infections in seven Ag-RDTs per SARS-CoV-2 log_10_ RNA copies per milliliter, performed in duplicates. Infectious virus was detected from all patient specimens unless marked with an asterisk (*, no infectious virus isolated).

**FIG 3:**
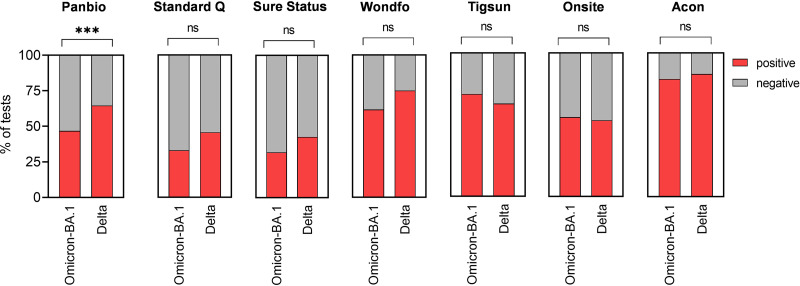
Percentage of positive/negative results for Omicron-BA.1 and Delta vaccine breakthrough infections per number of tests performed (Omicron-BA.1, *n* = 118; Delta, *n* = 108). ns, nonsignificant.

Testing with clinical specimens was done in duplicates for each specimen using seven Ag-RDTs to compare the performance for Omicron-BA.1 and Delta infections (Fig. 2). When assessing overall test positivity, for Omicron-BA.1 497/826 (60.17%) of tests showed a positive result compared to 489/756 (64.68%) (*z* = −1.85, *P* = 0.064) for Delta. Of 413 test pairs (test pair means two antigen tests conducted on a single participant), 27 showed a discordant result for Omicron-BA.1 versus 28 in 378 test pairs performed for Delta (*z* = 0.48, *P* = 0.631). When comparing overall sensitivity for Delta versus Omicron-BA.1 for each Ag-RDT, all Ag-RDTs showed comparable sensitivity performance (Table 2 and Fig. 3). Sensitivity in our specimen panels ranged from 39% to 85.6% for Omicron-BA.1 and from 45.4% to 87% for Delta, confirming the high variability of sensitivity between the different tests that was observed in our testing (Table 2).

**TABLE 2:** Detailed sensitivity for the seven Ag-RDTs tested with clinical samples

Ag-RDT	Sensitivity (%)		*P* ^*a*^
	Delta (*n* = 108)	Omicron-BA.1 (*n* = 118)	
Panbio	68.5	46.6	0.503
Standard Q	45.4	39.8	0.804
Sure Status	47.2	39.0	0.746
Onsite	77.8	68.6	0.735
Wondfo	68.5	75.4	0.833
Tigsun	58.3	59.3	0.944
Acon	87.0	85.6	0.946

a *P* values for logistic mixed-effect models (with tests nested into patients) are reported.

In this study, we sought to assess sensitivity with samples that demonstrated similar characteristics such as viral load, presence of infectious virus, and time since DPOS. Consistent with characteristics used in this study, we observed that the sensitivities of the Ag-RDTs were better with samples collected between 0 and 3 DPOS with a range of sensitivity values from 45.2% to 96.4% for Omicron-BA.1 and from 56.3% to 92.5% for Delta. In fact, the highest sensitivity of Omicron-BA.1 detection was observed with Acon (96.4%) and Wondfo (89.3%) Ag-RDTs and the lowest sensitivity of detection was observed with Sure Status (45.2%) and Standard Q (47.6%) Ag-RDTs. In contrast, the highest sensitivity of Delta detection was observed with Acon (92.5%), Onsite (85%), and Wondfo (80%) and the lowest sensitivity of detection was observed with Standard Q (56.3%). It should be highlighted that the infectious viral load in these samples was between 0 and 13,000 PFU/mL (cycle threshold [*C_T_*] values between 29.52 and 16.59) in Omicron-BA.1 breakthrough samples and between 0 and 16,000 PFU/mL in Delta breakthrough samples (*C_T_* values between 27.64 and 15.91) (Table 3 and see Table S2 in the supplemental material). Samples collected between 4 and 5 DPOS showed lower performance with a sensitivity ranging from 20.6% (Standard Q) to 58.8% (Acon) for Omicron-BA.1 with an infectious viral load ranging from 0 to 7,633 PFU/mL (*C_T_* values between 29.52 and 21.38) and a sensitivity ranging from 10.7% (Sure Status) to 71.4% (Acon) for Delta with an infectious viral load ranging from 0 to 440 FFU/mL (*C_T_* values between 32.95 and 22.44) (Table 3 and Table S2).

**TABLE 3:** Detailed sensitivity for the seven Ag-RDTs tested with clinical samples at 0 to 3 DPOS and 4 to 5 DPOS

Ag-RDT	Sensitivity (%)					
	DPOS 0–3			DPOS 4–5		
	Delta (*n* = 80)	Omicron-BA.1 (*n* = 84)	*P* ^*a*^	Delta (*n* = 28)	Omicron-BA.1 (*n* = 34)	*P* ^*a*^
Panbio	73.8	60.7	0.159	53.6	35.3	<0.001
Standard Q	56.3	47.6	0.307	14.3	20.6	0.913
Sure Status	60.0	45.2	<0.001	10.7	23.5	0.812
Onsite	85.0	76.2	0.781	57.1	50.0	0.600
Wondfo	80.0	89.3	0.763	35.7	41.2	0.937
Tigsun	68.8	67.96	0.973	28.6	38.2	0.203
Acon	92.5	96.4	0.807	71.4	58.8	0.744

a *P* values for logistic mixed-effect models (with tests nested into patients) are reported (except Panbio DPOS 0 to 3 and Tigsun DPOS 4 to 5, for which logistic regressions without tests were nested into patients because of convergence problems).

## DISCUSSION

Newly emerging variants necessitate a rapid assessment of the performance of diagnostic tests in use, particularly when there are mutations in the target gene. Here, we have performed a comprehensive laboratory-based evaluation study of eight Ag-RDTs with cultured Omicron-BA.1 virus as well as a retrospective clinical validation with 113 patient specimens.

Overall, we have observed a lower sensitivity to cultured Omicron-BA.1 virus across different Ag-RDTs than to earlier variants, suggesting that the virus itself is detected with lower sensitivity than other variants. We have observed differences between Ag-RDTs from different manufacturers, but also between assessments for PFU and RNA copy numbers. Reasons are most likely due to different ratios between numbers of infectious particles and RNA copies among the different SARS-CoV-2 variants. Since the main public health benefit of Ag-RDTs is the detection of individuals shedding infectious virus and not just viral RNA, assessment of infectious viral particles is of higher relevance in this context, and an overall tendency toward lower sensitivity was seen for both assessments. In the infectious virus analysis, Omicron-BA.1 has in general lower sensitivity than the previous VOCs Alpha, Beta, Gamma, and Delta, which were mainly detected with sensitivity comparable to that for B.1.610 (pre-VOC SARS-CoV-2). Notably, Omicron-BA.1 is the first VOC demonstrating a trend across most Ag-RDTs toward lower analytical sensitivity across assays.

Omicron-BA.1 has additional mutations in the nucleocapsid, some of which have been previously observed in circulating SARS-CoV-2, such as R203K and G204R in the Alpha VOC. Other mutations have not been observed or were only rarely observed in circulating SARS-CoV-2, so their impact on Ag-RDT performance is unknown. The virus isolate used in our study carries all four of the known nucleocapsid mutations (P13L, Del31-33, R203K, and G204R), confirmed from both patient specimens and virus isolate. The percentages of Omicron-BA.1 sequences with these mutations are 96.4% for P13L, 94.3% for Del31-33, 98.3 for R203K, and 98.3% for G204R of currently available Omicron-BA.1 sequences (18). As not all circulating Omicron-BA.1 lineages harbor all mutations, additional analysis with such isolates would be of interest; however, at the time of this study, no such isolates were available. However, our isolate represents the major circulating Omicron-BA.1 lineage.

In our clinical validation, we saw considerable heterogenicity between Ag-RDTs, with a nonsignificant loss of sensitivity for all Ag-RDT Omicron-BA.1 specimens. Comparisons of diagnostic assays by using different patient specimen collections are not trivial, and we have aimed for similar characteristics for the main determinants for rapid test performance, which are viral load, presence of infectious virus, and DPOS (19, 20).

Furthermore, we had access to detailed clinical data, and all specimens were from previously mRNA-vaccinated individuals, followed by a Delta or Omicron-BA.1 breakthrough infection. At least in most high-income countries with high vaccination rates, this group of individuals comprises the majority of Omicron-BA.1 infections observed, and therefore, our results are of immediate public health interest (21).

Few data are available so far on Ag-RDT performance for Omicron-BA.1 case detection. A small number of studies are available to date based on analytical performance and retrospective testing, but with differences in methodology and type of Ag-RDTs performed, and there are even fewer clinical validations.

One study used an approach similar to our study with somewhat different results: when comparing cultured virus isolates by viral RNA copy number, no difference in sensitivity was observed; however, in clinical specimens, 10-fold-higher viral loads were required for Omicron-BA.1 than for Delta to reach the same limit of detection in 50% of specimens (22). Results for detection of true-positive Omicron-BA.1 samples were higher in the category with low RT-PCR cycle threshold (*C_T_*) values/high viral load (*C_T_*, <25) and dropped drastically for *C_T_* values between 25 and 30. Confounders leading to partly different results could be largely methodology that varied between studies and could be due to a different set of Ag-RDTs investigated here.

In line with this and other previous findings, Ag-RDT sensitivity increased in parallel with decreasing SARS-CoV-2 *C_T_* values. Interestingly, consistent with the selected characteristics, we observed that the sensitivities of all Ag-RDTs used in this work increased when considering specimens collected at 0 to 3 DPOS, while those collected on days 4 to 5 had lower sensitivities (Table 3). A recent report from the U.S. Food and Drug Administration (FDA) announced that early data suggest reduced sensitivity for Omicron-BA.1, in line with our findings, although in their primary data, they used other Ag-RDT kits (23). A study performed by Public Health England (PHE) with cultured isolates of Omicron-BA.1 and wild-type SARS-CoV-2 across dilutions ranging from 12.5 to 1,250 focus-forming units/mL and from 30,000 to 4,070,000 viral copies did not find a loss in sensitivity for five Ag-RDTs (24). Only one of the Ag-RDTs validated here, the Acon Ag-RDT, was also validated in our study. In our analytical testing, reduced sensitivity was seen for Omicron-BA.1 compared to wild-type SARS-CoV-2 in this test, but we did not see a significant difference in the clinical specimens compared to Delta. Overall, in both our assessments, the Acon Ag-RDT was the most sensitive Ag-RDT for most variants, including Omicron-BA.1. Another study used two nasal swab samples each from Omicron-BA.1- and Delta-infected individuals and validated the Abbott Binax Now Ag-RDT, a test that was not included in our study (25). The authors of that study conclude that Omicron-BA.1 can be detected by this test, although no extensive validation for sensitivity was performed. For the same test, data from a single clinical validation study are available from an outpatient testing center in the United States using nasal swabs (26). Sensitivity of a single antigen test was 95.2% for individuals with a *C_T_* value of <30, indicating good sensitivity with high viral load. A high failure rate was observed when oral specimens (cheek swabs) were used.

The strength of our study is that we have validated eight and seven Ag-RDTs side by side for analytical and retrospective clinical sensitivity, respectively. Our selection of Ag-RDTs covers all of the three Ag-RDTs on the WHO-EUL and three others that are on the WHO-EUL approval waiting list and are thus of high global public health relevance (27, 28). If the lower sensitivity toward Omicron-BA.1 that we observed here is confirmed by findings from clinical validations at the point of care, the use of Ag-RDTs, especially in the early symptomatic period of an Omicron-BA.1 infection or in asymptomatic patients, could be less reliable. This may have important implications for public health measures. As our evaluation here was rather focused at the lower end of detection, results might be of higher relevance to testing in an asymptomatic population or in the very early infection phase, but not necessarily in the acute symptomatic infection phase when peak viral loads are reached.

Our study has several limitations. For cultured virus, the ratio between infectious virus, viral protein, and RNA copy number might differ considerably from the original human specimens. Samples in the retrospective testing have been submerged in viral transport medium, whereas the recommended sample type for Ag-RDT use is fresh swabs. This has introduced an extra dilution factor as well as an additional freeze-thaw cycle. Although we tried to keep the number of freeze-thaw cycles to a minimum, we cannot exclude loss of RNA, protein, or infectious virus, thus not reflecting fully the characteristics of a fresh patient specimen. To correct for loss of RNA after the first freeze-thaw cycle, we have retested viral RNA loads by RT-PCR and have used these values for comparison. Another limitation is that to compare across assays we have used the same approach as we did for analytical testing, with only 5 μL of the original patient viral transport medium (VTM) added to the buffer of each kit to be able to use the same specimens for testing with a high number of tests in parallel. The volume of viral transport medium added to the buffer was lower than what was recommended by some manufacturers, and for most Ag-RDTs, there was no recommendation on the use of swab samples in VTM. Therefore, viral loads of the original sample and sensitivities observed in our sample collection cannot be compared to results obtained from clinical validations performed on fresh samples, and our results should be interpreted as a comparison between Ag-RDTs and not as sensitivity thresholds for absolute viral loads and/or presence of infectious virus. Rather, we have investigated the lower end of sensitivity in the Ag-RDTs tested. Therefore, a reduced sensitivity in some tests, but not complete failure to detect Omicron-BA.1, could be of higher relevance at the beginning of the infection, when viral loads are still on the rise, and of less relevance once peak viral loads are reached. Another limitation is that we did not have information on potential previous infections of the patient from whom samples were tested, which could be an additional confounder due to the presence of mucosal immunity.

The lower sensitivities observed in this study could be due to a variant-specific impact on Ag-RDT performance. However, since many Omicron-BA.1 infections are currently observed in vaccinated individuals, it remains unclear if virus shedding and test performance differ between vaccinated and unvaccinated individuals, and no studies are available yet that investigate Ag-RDT performance in unvaccinated versus vaccinated individuals. To date, most validation studies of Ag-RDTs were done in the first year of the pandemic, before circulation of VOCs, and in mostly immune-naive individuals experiencing their primary SARS-CoV-2 infection. Other factors, such as *in vivo* shedding of infectious virus and overall viral load, can be one reason for differences in test performance.

Indeed, we have shown recently that both RNA viral loads and infectious titers in delta breakthrough infections led to significantly higher infectious titers than Omicron-BA.1 breakthrough, and thus, differences in viral load are likely to be the reason for lower sensitivity to Omicron-BA.1 in some tests (29).

Importantly, while analytical and retrospective testing may be a proxy for clinical sensitivity, it is not a replacement for clinical evaluations at the point of care. The discrepancies in our results between testing with cultured virus and that with retrospective patient samples highlight the need for proper clinical studies in well-defined patient cohorts. Also, the discrepancies observed between studies show that standardized protocols for such validations would be an asset to the scientific community, and it would be beneficial if manufacturers would give recommendations on how to use the test for retrospective testing.

Studies on diagnostic accuracy of Ag-RDTs performed at the point of care for the newly emerged VOC Omicron-BA.1 are urgently needed to guide public health responses.

## MATERIALS AND METHODS

### Clinical specimens.

One hundred thirteen nasopharyngeal swabs for diagnostics of SARS-CoV-2 by RT-PCR collected from symptomatic individuals in the outpatient testing center of the Geneva University Hospital and conserved in viral transport medium (VTM; Liofilchem) were included in this study. Infection with SARS-CoV-2 was initially diagnosed in the unit of the virology laboratory of the hospital by RT-PCR assay (Cobas 6800; Roche) followed by screening for S gene target failure (SGTF) with the TaqPath COVID-19 assay (Thermo Fisher). Remaining samples were stored at −80°C, usually on the same day or within 24 h. All samples had one freeze-thaw cycle before inoculation on cell cultures for infectious virus and for viral RNA quantification. For the majority of specimens, the direct Ag-RDT detection was performed at the same time. Due to logistical constraints, a subset of specimens had one additional freeze-thaw cycle for Ag-RDT testing only.

### Virus isolates.

The national reference center for emerging viruses at the laboratory of virology is hosted by the University Hospitals of Geneva (HUG). The laboratory is participating and coordinating the SARS-CoV-2 variant and genomic surveillance with ongoing full-genome sequencing of SARS-CoV-2-positive patient samples using Illumina NovaSeq. Each genome was analyzed with the Pangolin software to define the lineage of the variant. From each variant that falls into any of the categories of VOCs, at least one virus isolate is generated. Each SARS-CoV-2 variant was isolated from a single clinical sample. Isolates were grown in Vero-E6 cells and sequenced to ensure that no additional mutations were acquired as described previously (15). The Omicron-BA.1 variant was initially isolated on Vero-TMPRSS cells and then further passaged with a stock passage (p2) prepared on Vero-E6 cells. Vero-TMPRSS cells were kindly received from the National Institute for Biological Standards and Controls (NIBSC; catalog no. 100978). The following mutations and deletion in the nucleocapsid were present in the original patients’ sequences as well as in the virus isolate of the passage used in this study: R203K, G204R, P13L, and Del31-33.

### Viral load quantification.

*C_T_* values for E-gene target in each sample were determined by quantitative real-time reverse transcription PCR (RT-qPCR) using the SuperScript III Platinum one-step qRT-PCR kit (Invitrogen) after thawing. An *in vitro*-transcribed RNA standard for the E-gene assay was used for quantification of genome copy numbers (30). Presence of infectious virus was determined by focus-forming assay in Vero-E6 or Vero-TMPRSS cells as described previously (29). Briefly, patient samples were serially diluted and applied on a monolayer of cells. At 24 h postinoculation, cells were fixed and stained with a primary monoclonal antibody targeting SARS-CoV-2 nucleocapsid protein (Geneva Antibody Facility; JS02), following staining with secondary peroxidase-conjugated secondary antibody (Jackson ImmunoResearch; 109-036-09). Foci were visualized using True Blue horseradish peroxidase (HRP) substrate, and virus titers were counted as focus-forming units per milliliter (FFU/mL).

### Ag-RDT performance.

The 8 commercially available Ag-RDT products used in the study are summarized in Table S1 in the supplemental material. All Ag-RDTs target the nucleocapsid protein.

**(i) Analytical testing with cultured virus.** Each isolate has undergone serial dilutions at 1:2 in Dulbecco modified Eagle medium (DMEM). For each variant, we started the dilutions with the same virus concentration at 1.72E+04 PFU/mL. All Ag-RDT assays were performed according to the manufacturers’ instructions except that viral dilutions were added to the buffer instead of a swab specimen. All dilutions used for validation additionally were tested and quantified by RT-PCR assay for SARS-CoV-2 RNA copy numbers per milliliter. Each Ag-RDT kit has its respective buffer. For each serial dilution of each variant, 5 μL of dilution was applied in duplicate to the respective buffer of each kit and then applied to the Ag-RDT using only the materials provided in the kit under biosafety level 3 (BSL-3) conditions (16).

**(ii) Performance testing with clinical specimens.** For testing with clinical specimens, 5 μL of VTM of each specimen was directly added to the proprietary buffer and then applied to the Ag-RDT. Ag-RDT buffer without virus was used as a negative control. All Ag-RDT assays were read visually in duplicate. All visible bands were considered a positive result.

### Statistics.

We first compared whether log_10_ SARS-CoV-2 RNA copies, days post-symptom onset (DPOS), and presence of infectious disease were significantly different between the Delta and Omicron-BA.1 patients using simple linear and logistic regressions. We then tested whether the overall sensitivities and discordances differed between Delta and Omicron-BA.1 using proportion tests. Finally, we compared sensitivities for Delta and Omicron-BA.1 tests separately for each Ag-RDT. To take into account that each patient had two independent tests, we used mixed-effect logistic regressions with tests nested into patients. Data were analyzed using RStudio version 4.1.2.

### Ethical approval.

Ethical approval for samples used in this study for virus isolation was waived by the local ethics committee of the Geneva University Hospitals (HUG) that approves the usage of anonymized leftover patient samples collected for diagnostic purposes in accordance with our institutional and national regulations. The part of the study using patient specimens linked to clinical data (retrospective testing) was approved by the cantonal ethics committee (CCER Nr. 2021-01488). For this part, all study participants and/or their legal guardians provided informed consent.
