# Supplementary material for: Analytical Sensitivity of Eight Different SARS-CoV-2 Antigen-Detecting Rapid Tests for Omicron-BA.1 Variant
Source: Microbiol Spectr. 2022 Aug 8;10(4):e00853-22. doi: 10.1128/spectrum.00853-22 (PMC9430749; doi:10.1128/spectrum.00853-22)
Supplement: Supplemental file 1 — Supplemental material. Download spectrum.00853-22-s0001.pdf, PDF file, 0.1 MB [file spectrum.00853-22-s0001.pdf]

Supplementary material

Tables

Table S1. Overview of Ag-RDTs kits evaluated in the study.

	Name of kit	Manufacturer	Target protein	Approved by WHO
I	Panbio, COVID-19 Ag Rapid test device	Abbott	Nucleocapsid	Yes
II	Standard Q COVID-19 Ag	SD BIOSENSOR (Roche)	Nucleocapsid	Yes
III	Sure Status	Premier Medical Corporation	Nucleocapsid	Yes
IV	2019-nCoV Antigen test	Wondfo	Nucleocapsid	/
V	Beijng Tigsun Diagnostics Co. Ltd	Tigsun	Nucleocapsid	/
VI	CTK biotech	Onsite	Nucleocapsid	Yes
VII	ACON biotech	Flowflex	Nucleocapsid	Yes
VIII	NowCheck Covid- 19 Ag test	Bionote	Nucleocapsid	/

Table S2

Raw data for all infectious and RNA vial load used in this retrospective study. a) Delta breakthrough samples and b) Omicron-BA.1 breakthrough samples. Viral load CT Superscript: Ct values measured from thawed nasopharyngeal swab samples; DPOS: days post onset of symptoms; FFU/mL: iiter in focus forming units (FFU/ml).

a)

Sample number	CT-ORF1, Cobas-6800	Viral load Ct Superscript, BioRad	Infectious virus	DPOS	Titer FFU/ml
**01**	16	18.26	+	2	7000
**02**	16.5	19.65	+	0	7000
**03**	16.9	18.00	+	1	500.00
**04**	17.2	15.91	+	1	16000
**05**	17.3	21.27	+	2	1200.00
**06**	17.9	18.72	-	3	0.00
**07**	18	19.44	-	2	0.00
**08**	18.7	21.10	+	3	2000
**09**	18.8	22.56	+	3	1500
**10**	18.9	19.58	+	1	4000
**11**	19	22.76	+	3	2.70E+03
**12**	19.3	22.23	+	1	1750
**13**	19.4	23.68	+	3	200
**14**	19.4	21.37	+	2	1600

**15**	19.6	21.81	+	2	5000.00
**16**	19.6	20.40	+	1	1650.00
**17**	19.8	25.10	+	2	2550.00
**18**	19.8	22.61	+	0	4450
**19**	20	27.34	+	0	120
**20**	20	24.08	-	3	0.00
**21**	20.1	22.68	+	4	600
**22**	21.1	27.80	+	3	320
**23**	20.4	22.44	+	5	20
**24**	20.6	20.73	+	3	1900
**25**	20.7	23.46	+	2	1700.00
**26**	20.7	22.58	+	1	700.00
**27**	21.1	23.72	+	5	800
**28**	21.2	27.06	+	4	10
**29**	21.4	27.90	+	0	200
**30**	21.4	22.07	+	0	3000.00
**31**	21.5	21.66	+	0	300
**32**	21.6	25.94	+	3	40.00
**33**	21.9	26.56	+	5	190.00
**34**	21.9	24.26	-	2	0.00
**35**	21.9	24.23	+	2	600.00
**36**	22.3	27.54	-	0	0.00
**37**	22.4	26.88	-	4	0.00
**38**	22.6	28.27	+	3	600
**39**	22.7	27.10	+	5	195
**40**	23.1	24.58	+	4	190
**41**	23.3	28.95	-	4	0.00
**42**	23.4	29.24	-	5	0.00
**43**	23.5	27.64	-	0	0.00
**44**	23.7	25.93	+	5	440
**45**	23.9	24.45	-	2	0.00
**46**	25	31.31	-	4	0.00
**47**	25.8	29.35	-	5	0.00
**48**	26	32.95	-	5	0.00
**49**	19.7	22.11	+	2	405
**50**	20.70	22.36	+	3	40
**51**	20.8	22.37	+	2	120
**52**	21.4	25.82	-	2	0.00
**53**	21.6	26.13	-	1	0.00
**54**	22	24.69	+	2	20

b)

**Sample number**	**CT-ORF1, Cobas-6800**	**Ct Superscript, BioRad**	**Infectious virus**	**DPOS**	**FFU/ml**

**55**	14.6	16.59	+	1	13000
**56**	17.1	21.27	+	2	500
**57**	17.5	22.76	+	3	10
**58**	17.9	23.42	+	1	85
**59**	18.3	20.60	+	2	10
**60**	18.5	21.78	+	4	120
**61**	18.8	23.56	+	3	65
**62**	18.7	19.86	+	0	1600
**63**	19	20.09	+	2	155
**64**	19.1	21.25	+	3	2000
**65**	19.4	21.20	+	3	1400
**66**	19.7	21.48	+	0	285
**67**	19.9	21.38	+	5	355
**68**	20.2	22.99	+	2	60
**69**	20.2	22.07	+	2	220
**70**	20.2	22.96	+	2	115
**71**	20.3	23.04	+	5	110
**72**	20.7	22.81	+	1	325
**73**	20.7	21.59	+	0	600
**74**	20.8	22.50	+	2	40
**75**	20.8	27.67	-	5	0
**76**	21	25.13	+	3	30
**77**	21.1	22.23	+	2	100
**78**	21.1	23.51	+	2	120
**79**	21.4	25.09	+	4	65
**80**	21.4	22.80	+	1	20
**81**	21.6	24.99	-	2	0
**82**	21.2	26.62	+	3	30
**83**	22.3	17.78	+	3	60
**84**	22.3	26.87	-	5	0
**85**	22.7	24.53	+	2	70
**86**	22.7	25.55	-	2	0
**87**	22.8	25.03	+	2	60
**88**	22.9	23.70	+	4	10
**89**	23	22.18	-	5	0
**90**	23.2	24.99	+	2	55
**91**	23.4	25.67	+	4	30
**92**	23.5	26.12	-	4	0
**93**	23.9	28.07	+	3	10
**94**	24	23.08	+	4	20
**95**	24.2	27.11	-	4	0
**96**	24.5	27.36	-	4	0
**97**	24.7	26.45	+	4	7633.333

**98**	24.7	26.19	+	2	60
**99**	24.9	27.88	-	4	0
**100**	25.6	29.07	-	1	0
**101**	26.7	29.52	-	5	0
**102**	18.5	17.74	+	0	7500
**103**	18.8	21.09	+	1	75
**104**	21.7	22.99	+	1	40
**105**	19.4	22.16	+	1	800
**106**	20.2	21.23	-	2	0
**107**	19.1	23.61	+	2	395
**108**	18.5	19.77	+	2	600
**109**	19.7	22.17	+	2	150
**110**	18.6	21.30	+	3	260
**111**	20	26.55	-	3	0
**112**	22.2	25.16	+	3	140
**113**	20	21.91	+	4	400
